# Costs of cancer attributable to excess body weight in the Brazilian public health system in 2018

**DOI:** 10.1371/journal.pone.0247983

**Published:** 2021-03-11

**Authors:** Ronaldo Corrêa Ferreira da Silva, Luciana Ribeiro Bahia, Michelle Quarti Machado da Rosa, Thainá Alves Malhão, Eliane De Paula Mendonça, Roger dos Santos Rosa, Denizar Vianna Araújo, Luciana Grucci Maya Moreira, Arthur Orlando Correa Schilithz, Maria Eduarda Leão Diogenes Melo

**Affiliations:** 1 Brazilian National Cancer Institute José Alencar Gomes da Silva (INCA), Rio de Janeiro, Brazil; 2 Institute for Health Technology Assessment, State University of Rio de Janeiro, Rio de Janeiro, Brazil; 3 Social Medicine Department, Institute for Health Technology Assessment, Federal University of Rio Grande do Sul, Porto Alegre, Brazil; 4 Internal Medicine Department, Institute for Health Technology Assessment, State University of Rio de Janeiro, Rio de Janeiro, Brazil; 5 Department of Basic and Experimental Nutrition, Nutrition Institute, State University of Rio de Janeiro, Rio de Janeiro, Brazil; University of South Florida, UNITED STATES

## Abstract

**Objectives:**

The prevalence of excess body weight (EBW) has increased over the last decades in Brazil, where 55.4% of the adult population was overweight in 2019. EBW is a well-known risk factor for several types of cancer. We estimated the federal cost of EBW-related cancers in adults, considering the medical expenditures in the Brazilian Public Health System.

**Methods:**

We calculated the costs related to 11 types of cancer considering the procedures performed in 2018 by all organizations that provide cancer care in the public health system. We obtained data from the Hospital and Ambulatory Information Systems of the Brazilian Public Health System. We calculated the fractions of cancer attributable to EBW using the relative risks from the literature and prevalence from a nationally representative survey. We converted the monetary values in Reais (R$) to international dollars (Int$), considering the purchasing power parity (PPP) of 2018.

**Results:**

In Brazil, the 2018 federal cost for all types of cancers combined was Int$ 1.73 billion, of which nearly Int$ 710 million was spent on EBW-related cancer care and Int$ 30 million was attributable to EBW. Outpatient and inpatient expenditures reached Int$ 20.41 million (of which 80% was for chemotherapy) and Int$ 10.06 million (of which 82% was for surgery), respectively. Approximately 80% of EBW-attributable costs were due to breast, endometrial and colorectal cancers.

**Conclusion:**

A total of 1.76% of all federal cancer-related costs could be associated with EBW, representing a substantial economic burden for the public health system. We highlight the need for integrated policies for excess body weight control and cancer prevention.

## Introduction

The prevalence of excess body weight (EBW), including overweight and obesity, has increased alarmingly over the last few decades in many countries [1, 2]. This increase could be due to changes in the global food system, which promote energy-dense and nutrient-poor foods. In Brazil, a nationwide survey showed that the self-reported prevalence of EBW increased by more than 10% between 2006 and 2019, reaching 55.4% of the adult population in 2019 [3]. This condition is a well-known risk factor associated with several adverse health outcomes and an increased risk of many cancer types [4, 5].

In Brazil, the most frequent cancers in men are prostate, lung, colorectal, stomach and oral cavity cancer, and those in women are breast, colorectal, cervix, lung, and thyroid cancer [6]. The projection indicates that the absolute number of cancer cases attributable to EBW will double in 2025 compared to those in 2012, probably due to the aging of the population and the increasing prevalence of obesity [7]. There is strong evidence that EBW increases the risk of at least 12 types of cancer: breast (postmenopausal), colorectal, endometrium, oesophagus (adenocarcinoma), gallbladder, kidney, liver, mouth/pharynx/larynx, ovary, pancreas, prostate (advanced) and stomach (cardia) [8].

Cancer is a highly prevalent disease responsible for an economic burden with high direct (complex medical treatments) and indirect (loss of productivity, premature death) costs [9]. In 2016, the global cancer burden resulted in 213.2 million (95% uncertainty interval, 208.5–217.6 million) disability-adjusted life-years (DALYs), of which 98% came from years of life lost (YLLs) [10]. In 2010, the estimates indicated the global annual economic cost of cancer at approximately US$ 1.16 trillion [11]. In Brazil, the total cost of cancer (including mortality and morbidity) was estimated at Int$ 59.7 billion in 2015 and projected to reach Int$ 81 billion in 2020 [12]. The cost of mortality represents 63% of the total costs of cancer. Direct costs account for 20% ($10,025 per patient), and morbidity accounts for 17%. The Brazilian estimates of health expenditures were 9.5% of gross domestic product (GDP), with the average cost of cancer accounting for 1.7% of the GDP per year [12]. These results demonstrate the impact of cancer on society and the economy. Resource-driven strategies for prevention, early detection, and treatment could save up to 100 US$ billion in cancer treatment costs and prevent a loss of 2.4 to 3.7 million lives each year, with 80% of them in low/middle-income countries (LMICs) [11].

While high-income countries have inflated incidence rates of cancer, LMICs exhibit high mortality rates [13] and low global spending, with only 5% of global spending designated to these countries [14]. The economic burden of EBW-related cancer on LMIC healthcare remains to be quantified. The objective of this study was to estimate the federal cost of cancers in adults attributable to EBW. To accomplish this, we use information on the financial reimbursement of medical expenditures in the Brazilian Public Health System.

## Methods

### Data and study design

This study considered the Brazilian Public Health System (Sistema Único de Saúde—SUS) perspective as a payer and applied a top-down costing methodology. We calculated the costs for procedures performed in 2018 for individuals 30 years and older (for postmenopausal breast cancer, we considered women aged 50 years or older), considering the 2008 BMI prevalence of individuals 20 years and older who rely exclusively on the public health system. We also retrieved cost data from the Hospital Information System (SIH/SUS) and the Ambulatory Information System (SIA/SUS) available via an online open-access administrative database at the Brazilian Public Health System Data Processing Department (DATASUS) [15]. Both systems contain financial information on the procedures carried out on individuals with EBW-related cancers registered according to the 10th revision of the International Statistical Classification of Diseases and Related Health Problems (ICD-10) [16]. Summing all monetary values from each type of cancer according to its ICD-10 codes was used to calculate the total federal costs.

Estimating the federal costs of EBW-related cancers requires three types of data: 1. the estimates of relative risk (RR) for each EBW-related cancer; 2. the prevalence of EBW in Brazil; and 3. the total cost for each type of cancer. We used the first two to estimate the fractions of cancer attributable to EBW. We performed the analysis stratified by sex in endometrium, ovary, postmenopausal breast, and prostate cancers. For the other types, we considered the data for both sexes.

### Risk estimates and cancer sites

We obtained the RR for each cancer site from the World Cancer Research Fund (WCRF) and the American Institute for Cancer Research (AICR) Systematic Literature Reviews (SLR) (S1 and S2 References). We included cancer sites with strong evidence of association (convincing or probable) with EBW according to these organizations [17], except mouth/ pharynx/larynx cancer. We excluded this specified cancer because we verified the association only among nonsmokers, and there is no nationwide study of EBW prevalence stratified by smoking status in the analyzed period. We included the following cancer types: (1) breast (postmenopausal), (2) colorectal, (3) endometrium, (4) oesophagus (adenocarcinoma), (5) gallbladder, (6) kidney, (7) liver, (8) ovary, (9) pancreas, (10) prostate cancer (advanced), and (11) stomach (cardia) (S1 Table). Based on previous estimates from the Brazilian population, we assumed that 8.6% of all esophageal cancers were adenocarcinomas [18] and that 22% of all prostate cancers were advanced [19].

### Prevalence of EBW

We obtained the median body mass index (BMI) and prevalence rates for each excess weight category from the 2008–2009 National Household Budget Survey data conducted by the Brazilian Institute of Geography and Statistics (IBGE) [20]. We used the information on EBW from 2008 once scientific literature shows that a latency period of 10 years is a typical temporal gap from BMI measurement to incident cancer in systematic reviews. It is also an interval consistent with the beneficial effect of weight loss on subsequent cancer [21–23].

We considered the following weight categories: overweight (25≤BMI<30 kg/m^2^), class I obesity (30≤BMI<35 kg/m^2^), class II obesity (35≤BMI<40 kg/m^2^), and class III obesity (BMI≥40 kg/m^2^). The analysis performed in SAS Studio release 3.8 (Basic Edition) and R Studio version 1.3.1093 considered only individuals 20 years and older who exclusively use the public health system.

### Data analysis

#### Meta-analysis

We obtained the RR for each cancer site from systematic reviews that analyzed the association between EBW and cancer incidence. For ovary and prostate cancers, the summary RR of the WCRF/AICR Systematic Literature Review (SLR) [24, 25] included studies with mortality and cancer incidence outcomes. For this reason, we performed a new meta-analysis in STATA software version 13 using random-effects models considering only the incidence as the outcome (S1 Appendix).

#### Population attributable fractions (PAFs)

To estimate the PAFs of the general population for each type of cancer, we performed the following steps: (1) transform all RRs from 5 to 1 kg/m^2^ BMI increments to obtain the excess RR per unit of BMI [26]. We used the following equation: RR1 = exp(ln(RR5)/5), where RR1 represents the RR for one BMI unit and RR5 the RR for five BMI units (kg/m2) [26]; (2) assess the RRs of cancer for each BMI category using the equation (RRx = R^Mx-25^), where R represents the RR of cancer for one BMI unit (kg/m2) increase and Mx represents the median BMI for category x, where x represents each of the four BMI categories (overweight, obesity I, obesity II or obesity III) [27]; (3) measure the PAFs according to the following formula: (∑x Px (RRx—1))/(1+∑x Px (RRx—1)), where Px is the proportion of the population within each category x and RRx is the RR of cancer for each BMI category [28].

To estimate the 95% confidence intervals (CIs) for PAFs, we used the Monte Carlo simulation method [29–31]. We admitted the random values of the RR from a log-normal distribution considering its associated variance estimated from 95% CIs. We drew the prevalence values from a binomial distribution with parameter n as the number of survey participants and parameter p as the prevalence of exposure estimated from the survey. We simulated a total of 10,000 samples and used, as the lower and upper limits of its 95% CI, the 2.5th and 97.5th percentiles of the resulting PAF distribution. We conducted these analyses in R Studio version 1.3.1093.

#### Cost analysis

First, we estimated the total cost of cancer (related and nonrelated to EBW), and then we isolated the EBW-related cancer cost. To assess the costs of cancer attributable to EBW, we multiplied the total federal cost of each cancer by its corresponding PAF.

We grouped the federal costs as follows: (1) outpatient costs (chemotherapy, which included conventional chemotherapy, targeted therapy, hormone therapy, immunotherapy and supportive therapy; radiotherapy; and other ambulatory procedures, which included nuclear medicine and general costs in oncology), (2) inpatient costs (surgery; and other hospital costs, which included diagnostic and clinical procedures, and organ, tissue, and cell transplantation) and (3) total costs (outpatient plus inpatient costs).

We converted the monetary values in Reais (R$) to international dollars (Int$), considering the purchasing power parity (PPP) for the same year (conversion factor 2.03) [32] and conducted these analyses in the Tabwin program and Microsoft Excel Office^®^ 2007 spreadsheets.

The study was approved by the Institutional Review Board of the Brazilian National Institute of Cancer José Alencar Gomes da Silva (INCA) at the Brazilian Ministry of Health (MS) (CAAE 12008119.8.0000.5274).

## 3 Results

### RR and PAF

The RRs of cancer per 5 kg/m^2^ and 1 kg/m^2^ increase in BMI and the estimated PAFs of the 11 types of cancer included in this study are shown in Table 1. The PAF varied greatly by cancer site, ranging from 1.79% for colorectal cancer to 23.29% for endometrial cancer. The highest PAFs were estimated for cancer of endometrium (23.29%; 95% CI: 22.45–24.14%), oesophagus (adenocarcinoma, 16.05%; 95% CI: 15.24–16.86%) and liver (14.50%; 95% CI: 13.27–15.73%). The lowest PAFs were for colorectal cancer (1.79%; 95% CI: 1.64–1.93%), ovary (2.12%; 95% CI: 1.83–2.41%) and prostate (advanced, 2.42%; 95% CI: 2.06–2.79%).

**Table 1:** Relative risk (RR) and population attributable fraction (PAF) of excess body weight-associated cancers.

Type of cancer	RR per 5 kg/m^2^ increase in BMI (95% CI)	RR per 1 kg/m^2^ increase in BMI (95% CI)	PAF % (95% CI)
Breast (post-menopausal)^a^	**1.12** (1.09–1.15)	**1.02** (1.02–1.03)	**5.16** (4.92–5.41)
Colorectal^a^	**1.05** (1.03–1.07)	**1.01** (1.01–1.01)	**1.79** (1.64–1.93)
Endometrium^a^	**1.50** (1.42–1.59)	**1.08** (1.07–1.10)	**23.29** (22.45–24.14)
Gallbladder^a^	**1.23** (1.10–1.39)	**1.04** (1.02–1.07)	**7.96** (7.14–8.78)
Kidney^a^	**1.30** (1.25–1.36)	**1.05** (1.05–1.06)	**10.29** (9.83–10.77)
Liver^a^	**1.43** (1.19–1.70)	**1.07** (1.04–1.11)	**14.50** (13.27–15.73)
Oesophagus (adenocarcinoma)^a^	**1.48** (1.35–1.62)	**1.08** (1.06–1.10)	**16.05** (15.24–16.86)
Ovary^b^	**1.06** (1.02–1.10)	**1.01** (1.00–1.02)	**2.12** (1.83–2.41)
Pancreas^a^	**1.10** (1.07–1.14)	**1.02** (1.01–1.03)	**3.54** (3.29–3.80)
Prostate (advanced)^b^	**1.08** (1.03–1.14)	**1.02** (1.01–1.03)	**2.42** (2.06–2.79)
Stomach (cardia)^a^	**1.23** (1.07–1.40)	**1.04** (1.01–1.07)	**7.98** (7.05–8.93)

Abbreviations: RR; relative risk; CI, confidence interval; PAF, population attributable fraction.

^a^ RR was calculated by meta-analysis studies from WCRF/AICR SLR CUP Reports (S1 Reference).

^b^ RR was calculated by meta-analysis excluding mortality studies from WCRF/AICR SLR CUP Reports (S2 Reference).

### Cost

In Brazil, the 2018 federal cost of inpatient and outpatient care for all types of cancer was Int$ 1.73 billion, of which Int$ 710.09 million was for EBW-related cancer care. Of this amount, 4.29% (Int$ 30.48 million) was attributable to EBW (Table 2). Outpatient and inpatient costs reached Int$ 20.41 million (79% for chemotherapy, and 14% for radiotherapy) and Int$ 10.06 million (82% for surgery), respectively. Outpatient costs represented nearly 70% of the total costs attributable to EBW. Only gallbladder, kidney, stomach and liver cancers had inpatient costs higher than outpatient costs. Breast and endometrial cancer were responsible for the largest share of outpatient and inpatient EBW-attributable costs (Table 2).

**Table 2:** Federal costs of cancers attributable to excess body weight according to type of medical care. Brazil, 2018.

Cancer Type	Outpatient				Inpatient				Total			
	Total	Attributable to excess weight		Chemo/ Radio/ Other	Total	Attributable to excess weight		Surgery/Others	Total	Attributable to excess weight		Outpatient/Inpatient
	(Int $)	(Int $)	%	%	(Int $)	(Int $)	%	%	(Int $)	(Int $)	%	%
Breast^a^	227,567,362	11,744,822	5.16	80.69/16.52/ 2.79	47,533,147	2,453,200	5.16	88.72/11.28	275,100,509	14,198,023	5.16	82.72/17.28
Endometrium	11,682,030	2,720,263	23.29	27.83/65.73/ 6.44	10,442,596	2,431,650	23.29	89.66/10.34	22,124,626	5,151,914	23.29	52.80/47.20
Colorectal	173,538,409	3,100,488	1.79	82.57/5.36/ 12.07	95,255,960	1,701,871	1.79	77.74/22.26	268,794,369	4,802,358	1.79	64.56/35.44
Kidney	2,692,764	277,096	10.29	57.07/13.42/ 29.51	9,703,388	998,515	10.29	87.24/12.76	12,396,152	1,275,611	10.29	21.72/78.28
Prostate^b^	38,105,015	922,598	2.42	66.68/30.84/ 2.48	10,885,352	263,556	2.42	90.24/9.76	48,990,367	1,186,154	2.42	77.78/22.22
Liver	1,593,196	231,008	14.50	61.19/3.12/ 35.68	6,362,226	922,500	14.50	65.35/34.65	7,955,422	1,153,508	14.50	20.03/79.97
Pancreas	14,652,679	519,202	3.54	94.81/2.16/ 3.03	11,174,100	395,942	3.54	74.64/25.36	25,826,779	915,144	3.54	56.73/43.27
Ovary	20,682,447	437,605	2.12	95.86/0.63/ 3.51	18,718,158	396,044	2.12	91.53/8.47	39,400,605	833,649	2.12	52.49/47.51
Oesophagus^c^	1,433,569	230,152	16.05	54.64/41.78/ 3.58	1,218,912	195,690	16.05	63.94/36.06	2,652,481	425,842	16.05	54.05/45.95
Stomach^d^	2,110,018	168,481	7.98	85.32/7.16/ 7.53	2,165,205	172,888	7.98	88.44/11.56	4,275,223	341,369	7.98	49.35/50.65
Gallbladder	845,104	67,258	7.96	81.55/6.42/ 12.03	1,728,672	137,578	7.96	78.85/21.15	2,573,776	204,836	7.96	32.84/67.16
**All EBW cancers**	494,902,593	20,418,973	4.13	79.83/13.74/ 6.43	215,187,716	10,069,435	4.68	82.51/17.49	710,090,309	30,488,407	4.29	69.70/30.30
All cancers*	1,071,818,542	20,418,973	1.91	71.87/20.36/ 7.78	664,616,967	10,069,435	1.52	76.32/23.68	1,736,435,509	30,488,407	1.76	61.73/38.27

Abbreviations: EBW, excess body weight; Int $, international dollar; N, absolute number; % percentage; Chemo, chemotherapy; Radio, radiotherapy.

^a^. Postmenopausal breast cancer;

^b^. Advanced prostate cancer;

^c^. Oesophageal adenocarcinoma;

^d^. Stomach cardia cancer.

*Both *in situ* and invasive cancers (including nonmelanoma skin cancers).

There was a wide variation in costs by EBW-related cancers, ranging from Int$ 204.8 thousand (gallbladder cancer) to Int$ 14.1 million (breast cancer). Breast, endometrial, and colorectal cancers accounted for almost 80% of the total costs attributable to EBW. Costs due to kidney, prostate (advanced), liver, and pancreatic cancer add up to 15%. The remaining cancers (ovary, oesophagus, stomach, and gallbladder) were responsible for approximately 5% of costs (Table 3).

**Table 3:** Federal costs of cancer attributable to excess body weight and respective confidence intervals. Brazil, 2018.

Cancer Type	Outpatient Int$ (95% CI)	Inpatient Int$ (95% CI)	Total Int$ (95% CI)
Breast^a^	**11,744,822** (11,186,231–12,304,738)	**2,453,200** (2,336,525–2,570,153)	**14,198,023** (13,522,755–14,874,891)
Endometrium	**2,720,263** (2,622,155–2,820,171)	**2,431,650** (2,343,915–2,520,958)	**5,151,914** (4,966,029–5,341,128)
Colorectal	**3,100,488** (2,838,609–3,355,268)	**1,701,871** (1,558,125–1,841,721)	**4,802,358** (4,396,734–5,196,989)
Kidney	**277,096** (264,748–289,953)	**998,515** (954,019–1,044,846)	**1,275,611** (1,218,766–1,334,799)
Prostate^b^	**922,598** (784,394–1,062,792)	**263,556** (224,076–303,605)	**1,186,154** (1,008,470–1,366,397)
Liver	**231,008** (211,454–250,569)	**922,500** (844,413–1,000,617)	**1,153,508** (1,055,867–1,251,187)
Pancreas	**519,202** (481,360–556,247)	**395,942** (367,084–424,193)	**915,144** (848,444–980,439)
Ovary	**437,605** (377,841–498,436)	**396,044** (341,956–451,098)	**833,649** (719,798–949,534)
Oesophagus^c^	**230,152** (218,491–241,746)	**195,690** (185,775–205,548)	**425,842** (404,265–447,295)
Stomach^d^	**168,481** (148,783–188,418)	**172,888** (152,674–193,346)	**341,369** (301,457–381,765)
Gallbladder	**67,258** (60,332–74,206)	**137,578** (123,409–151,789)	**204,836** (183,741–225,995)

Abbreviations: Int $, international dollar; CI, confidence interval.

^a^. Postmenopausal breast cancer;

^b^. Advanced prostate cancer;

^c^. Oesophageal adenocarcinoma;

^d^. Stomach cardia cancer.

## 4 Discussion

Our study demonstrated that in 2018, slightly more than Int$ 30 million federal expenditures in cancer care were attributable to EBW. This value corresponded to 1.76% of the total costs of cancer care in the Brazilian Public Health System in 2018 (Int$ 1.73 billion). Breast, colorectal, and endometrial cancers represented almost 80% of the total costs attributable to EBW, with breast cancer contributing to nearly 50% of all cancer care costs. As noted in the results section, 80% of the total costs were due to three cancers (20% of causes), which is evidence of the Pareto principle. Unfortunately, only 5% of breast cancers and almost 2% of colorectal cancers are attributable to EBW, limiting the cost reduction of preventive interventions focusing on this cause. To the best of our knowledge, this is the first study to estimate cancer costs attributable to EBW using the attributable risk methodology in Brazil.

There is considerable variability among studies regarding the parameters used for population attributable fractions for EBW, generating different estimates and making it difficult to compare populations. In our study, PAFs varied from 1.79% (colorectal cancer) to 23.29% (endometrial cancer), representing a wide range. Previous studies demonstrated different estimates from 0.2% to 8% for all-cancer incidence [33]. Whiteman and Wilson (2016) surveyed and summarized the proportions of cancers attributable to modifiable causes across the world. For EBW-related cancers, the range of median PAFs reported for each cancer site was large (5% to 42.5%) [34] (S2 Table).

Costs varied according to cancer type and extent of the disease, depending on the need for less or more complex treatments. In our analysis, the largest share of costs attributable to EBW was outpatient chemotherapy, followed by inpatient surgery and outpatient radiotherapy. In Brazil, the shared outpatient and inpatient costs of all EBW cancers were 69.70% and 30.30%, respectively. Likewise, in 2014, USA showed a similar outpatient and inpatient share of total cancer costs: 73% of cancer costs by type of service were outpatient and 27% inpatient. This is quite different from European Union cancer costs in 2009, where 55% were inpatient costs and 45% were outpatient costs [35, 36].

Postmenopausal breast cancer was responsible for most of the costs, followed by endometrial and colorectal cancer. Except for endometrial cancer, we expected this result considering that postmenopausal breast and colorectal cancer are among the most prevalent cancer types in Brazil [6]. Endometrial cancer is the 8th most common cancer in Brazil, and EBW is so strongly associated with this type of cancer that it has the highest PAF of all investigated cancers. On the other hand, although prostate cancer is the second most prevalent cancer in our country, it did not appear to be one of the highest EBW-attributable costs. There is strong evidence that the association between EBW and this type of cancer occurred only for advanced cases. For this reason, we only considered advanced prostate (approx. 22% of total prostate cancers), resulting in a low attributable fraction. The proportion of health-care costs accounting for the most prevalent types of cancers varied substantially between countries in the European Union: breast cancer accounted for the highest health-care costs (€6.73 billion; 13% of all cancer-related health-care costs), followed by colorectal cancer (€5.57 billion; 11%), prostate cancer (€5.43 billion; 11%), and lung cancer (€4.23 billion; 8%) in 2009 [36].

Although the Brazilian Public Health System provides universal care to all citizens, nearly 75% of the population relies exclusively on it. Public health expenditures, as a percent of GDP, rose from 3.16% to 4.05% between 2003 and 2017. The proportional contribution of the federal government to total expenditure changed from 50.1% in 2003 to 43.2% in 2017 [37]. In all countries, especially LMICs, high cancer morbidity and mortality translate into a significant reduction in GDP [38]. While most funding for cancer care is directed toward developing new treatments, prevention often remains neglected. The price of new cancer medicines is growing substantially, and many health care systems are unable to support this demand. An adequate approach seems to integrate prevention and treatment since primary prevention could avert 30–50% of new cancer cases if modifiable risk factors are eliminated [38]. Interventions may target individuals and populations through policy implementation. We must tailor the prevention according to the local setting (epidemiological, technological, cultural, demographic, and economic context). One major challenge is to persuade decision-makers to invest in primary prevention since the benefits take time to materialize.

When limiting primary prevention initiatives, LMICs may not have sufficient resources to diagnose and treat all new cancer cases. The costs of cancer care may become unsustainable and could lead to inequities in access to treatment. Moreover, several chronic diseases, such as cancer, cardiovascular disease, diabetes, and some neurological conditions, share many risk factors, like unhealthy diet, obesity, and smoking. Thus, the primary prevention of these risk factors will have a significant impact on several noncommunicable diseases.

Interventions aiming to reduce the prevalence of EBW that focus on weight loss treatment could also, theoretically, decrease many types of cancer. However, studies regarding weight loss and subsequent changes in cancer risk are sparse. The Women’s Health Initiative Study demonstrated that intentional weight loss (decrease >5%), which occurred in 7.9% out of 58,667 postmenopausal women aged 50–79 in three-year follow-up, was associated with a 12% reduced risk of endometrial, breast, and colorectal cancer [39]. A systematic review of cohort studies identified the association between bariatric surgery and significant reductions in mortality, cardiovascular events, and cancer risk (mainly in women) [40]. More studies are needed to determine whether sustained weight loss can reduce the incidence of various EBW-related cancer types.

We should highlight some limitations of our study. First, the SIH/SUS and SIA/SUS databases provide information only on the total amount reimbursed by the federal government to Brazilian health services, which does not consider other federal funding or the state and municipal counterparts. Thus, our financial data were conservative and underestimated. As we used a secondary database, potential misclassification may have influenced the results. Finally, as we did not find Brazilian studies evaluating the association of EBW and cancer incidence, we used RRs from different populations to estimate the PAFs.

## 5 Conclusions

This study estimated the federal government cancer expenditures attributable to EBW in Brazil in 2018. Slightly more than Int$ 30 million (1.76%) out of Int$ 1.7 billion of federal cancer care costs were attributable to EBW, representing a substantial economic burden in Brazil. Our findings highlight the importance of investing in primary prevention strategies, especially in countries where financial resources are scarce, such as in Brazil. Quantifying the financial burden of cancer attributable to EBW can help policymakers prioritize cancer control policies. It is time to promote a public debate to seek adequate balance between what is spent on prevention, specifically on EBW prevention, and treatment of cancer.

## Supporting information

S1 Reference (DOCX)

S2 Reference (DOCX)

S1 Table List of International Classification of Diseases (ICD) codes. (DOCX)

S2 Table Comparison between the population attributable fraction (PAF) found in our study and in the Whiteman & Wilson (2016) review. (DOCX)

S1 Appendix (DOCX)
