# Supplementary material for: Costs of cancer attributable to excess body weight in the Brazilian public health system in 2018
Source: PLoS One. 2021 Mar 11;16(3):e0247983. doi: 10.1371/journal.pone.0247983 (PMC7951921; doi:10.1371/journal.pone.0247983)
Supplement: S1 Reference — (DOCX) [file pone.0247983.s001.docx]

**Supporting Information**

**Reference 1**

1. World Cancer Research Fund/American Institute for Cancer Research. Systematic literature review - The associations between food, nutrition and physical activity and the risk of Breast cancer. In: Continuous Update Project [Internet]. 2017 [cited 25 Oct 2019] p. 762. Available: https://www.wcrf.org/sites/default/files/breast-cancer-slr.pdf

2. World Cancer Research Fund/ American Institute for Cancer Research. Systematic Literature Review. The Associations between Food, Nutrition and Physical Activity and the Risk of Colorectal Cancer. In: Continuous Update Project [Internet]. 2017 [cited 25 Oct 2019] p. 1355. Available: https://www.wcrf.org/sites/default/files/colorectal-cancer-slr.pdf

3. World Cancer Research Fund/ American Institute for Cancer Research. Systematic Literature Review - The Associations between Food, Nutrition and Physical Activity and the Risk of Endometrial Cancer. In: Continuous Update Project. [Internet]. 2012 [cited 25 Oct 2019] p. 240. Available: https://www.wcrf.org/sites/default/files/endometrial-cancer-slr.pdf

4. World Cancer Research Fund/American Institute for Cancer Research. Systematic Literature Review - The Associations between Food, Nutrition and Physical Activity and the Risk of Oesophageal Cancer. In: Continuous Update Project [Internet]. 2015 p. 287. Available: https://www.wcrf.org/sites/default/files/Oesophageal-cancer-report.pdf

5. World Cancer Research Fund/American Institute for Cancer Research. Systematic Literature Review - The Associations between Food, Nutrition and Physical Activity and the risk of Kidney Cancer. In: Continuous Update Project [Internet]. 2015 [cited 25 Oct 2019] p. 258. doi:10.1007/s12082-007-0105-4

6. World Cancer Research Fund/American Institute for Cancer Research. Systematic Literature Review. The associations between food, nutrition and physical activity and the risk of Liver Cancer. In: Continuous Update Project [Internet]. 2015 [cited 25 Oct 2019] p. 114. Available: https://www.wcrf.org/sites/default/files/liver-cancer-slr.pdf

7. World Cancer Research Fund/American Institute for Cancer Research. Systematic Literature Review. The associations between food, nutrition and physical activity and the risk of Pancreatic Cancer. In: Continuous Update Project [Internet]. 2011 [cited 25 Oct 2019] p. 470. Available: https://www.wcrf.org/sites/default/files/pancreatic-cancer-slr.pdf

8. World Cancer Research Fund/American Institute for Cancer Research. Systematic Literature Review - The Associations between Food, Nutrition and Physical Activity and the Risk of Stomach cancer. In: Continuous Update Project [Internet]. 2018. Available: http://www.aicr.org/continuous-update-project/reports/stomach-cancer-report.pdf

9. World Cancer Research Fund/American Institute for Cancer Research. Systematic Literature Review. The associations between food, nutrition and physical activity and the risk of Gallbladder Cancer. In: Continuous Update Project [Internet]. 2014 [cited 25 Oct 2019] p. 48. Available: https://www.wcrf.org/sites/default/files/gallbladder-cancer-slr.pdf
