# Supplementary material for: Costs of cancer attributable to excess body weight in the Brazilian public health system in 2018
Source: PLoS One. 2021 Mar 11;16(3):e0247983. doi: 10.1371/journal.pone.0247983 (PMC7951921; doi:10.1371/journal.pone.0247983)
Supplement: S2 Reference — (DOCX) [file pone.0247983.s002.docx]

**Supporting Information**

**Reference 2**

1. World Cancer Research Fund/American Institute for Cancer Research. Systematic literature review - The associations between food, nutrition and physical activity and the risk of Ovarian Cancer. In: Continuous Update Project [Internet]. 2013 [cited 25 Oct 2010] p. 322. Available: https://www.wcrf.org/sites/default/files/ovarian-cancer-slr.pdf

2. World Cancer Research Fund/American Institute for Cancer Research. Systematic Literature Review. The Associations between Food, Nutrition and Physical Activity and the Risk of Prostate Cancer. In: Continuous Update Project [Internet]. 2014 [cited 25 Oct 2019]. Available: https://www.wcrf.org/sites/default/files/prostate-cancer-slr.pdf
