# Supplementary material for: Costs of cancer attributable to excess body weight in the Brazilian public health system in 2018
Source: PLoS One. 2021 Mar 11;16(3):e0247983. doi: 10.1371/journal.pone.0247983 (PMC7951921; doi:10.1371/journal.pone.0247983)
Supplement: S1 Table — (DOCX) [file pone.0247983.s003.docx]

**Supporting Information**

**Table 1.** List of International Classification of Diseases (ICD) codes

| **Cause** | **ICD-10** |
| --- | --- |
| Breast cancer^a^ | C50; D05 |
| Endometrial cancer | C54; D07.0 |
| Colorectal cancer | C18-C20; D01.0-D01.2 |
| Kidney cancer | C64-C65 |
| Prostate cancer | C61 |
| Liver cancer | C22.0; C22.9 |
| Pancreatic cancer^b^ | C25 |
| Ovarian cancer | C56 |
| Esophageal cancer^c^ | C15; D00.1 |
| Gastric cancer^d^ | C16.0 |
| Gallbladder cancer | C23 |

Abbreviations: ICD, International Classification of Diseases a. Postmenopausal breast cancer; b. Advanced prostate cancer; c. Oesophageal adenocarcinoma; d. Stomach cardia cancer.
