# Supplementary material for: Costs of cancer attributable to excess body weight in the Brazilian public health system in 2018
Source: PLoS One. 2021 Mar 11;16(3):e0247983. doi: 10.1371/journal.pone.0247983 (PMC7951921; doi:10.1371/journal.pone.0247983)
Supplement: S2 Table — (DOCX) [file pone.0247983.s004.docx]

**S1 Table 2.** Comparison between the population attributable fraction (PAF) found in our study and in the Whiteman & Wilson (2016) review

| **Type of cancer** | **PAF % (95% CI)**  **Our study** | **Median PAF % (Lowest/ Highest PAF)**  **Whiteman & Wilson (2016)** |
| --- | --- | --- |
| Breast (post-menopausal) | **5.16** (4.92 – 5.41) | Female: **10.0** (1.0 – 22.6) |
| Colorectal | **1.79** (1.64 – 1.93) | Male: **11.8** (6.6 – 35.4)  Female: **11.6** (4.8 – 20.8) |
| Endometrium | **23.29** (22.45 – 24.14) | Female: **36.0** (3.0 – 61.0) |
| Gallbladder | **7.96** (7.14 – 8.78) | Male: **11.0** (2.0 – 19.7)  Female: **42.5** (4.0 – 61.0) |
| Kidney | **10.29** (9.83 – 10.77) | Male: **14.0** (0.8 – 26.0)  Female: **24.5** (2.0 – 43.0) |
| Liver | **14.50** (13.27 – 15.73) | **-** |
| Oesophagus (adenocarcinoma) | **16.05** (15.24 – 16.86) | Male: **29.0** (2.0 – 49.0)  Female: **37.0** (3.0 – 59.0) |
| Ovary | **2.12** (1.83 – 2.41) | Female: **5.0** (1.0 – 10.0) |
| Pancreas | **3.54** (3.29 – 3.80) | Male: **8.0** (0.5 – 17.0)  Female: **8.4** (1.0 – 20.0) |
| Prostate (advanced) | **2.42**(2.06 – 2.79) | **-** |
| Stomach (cardia) | **7.98** (7.05 – 8.93) | **-** |

Abbreviations: CI, confidence interval; PAF, population attributable fraction.

Whiteman DC, Wilson LF. The fractions of cancer attributable to modifiable factors: A global review. Cancer Epidemiol. 2016;44: 203–221. doi:10.1016/j.canep.2016.06.013
