# Supplementary material for: Costs of cancer attributable to excess body weight in the Brazilian public health system in 2018
Source: PLoS One. 2021 Mar 11;16(3):e0247983. doi: 10.1371/journal.pone.0247983 (PMC7951921; doi:10.1371/journal.pone.0247983)
Supplement: S1 Appendix — (DOCX) [file pone.0247983.s005.docx]

**Supporting Information**

**Appendix:** Meta-analysis of ovary and prostate cancers by incidence as outcome.

A new meta-analysis was performed for ovarian and prostate cancers including only the studies with incidence outcome of the WCRF/AICR Systematic Literature Review (SLR)^1,2.^ It was used random-effects model since it allows that the *true* effect size might differ from study to study. This analysis was conducted in the software STATA version 13 using the *metan* command.

**Figure 1**. Forest plot of the incidence studies of ovarian cancer.


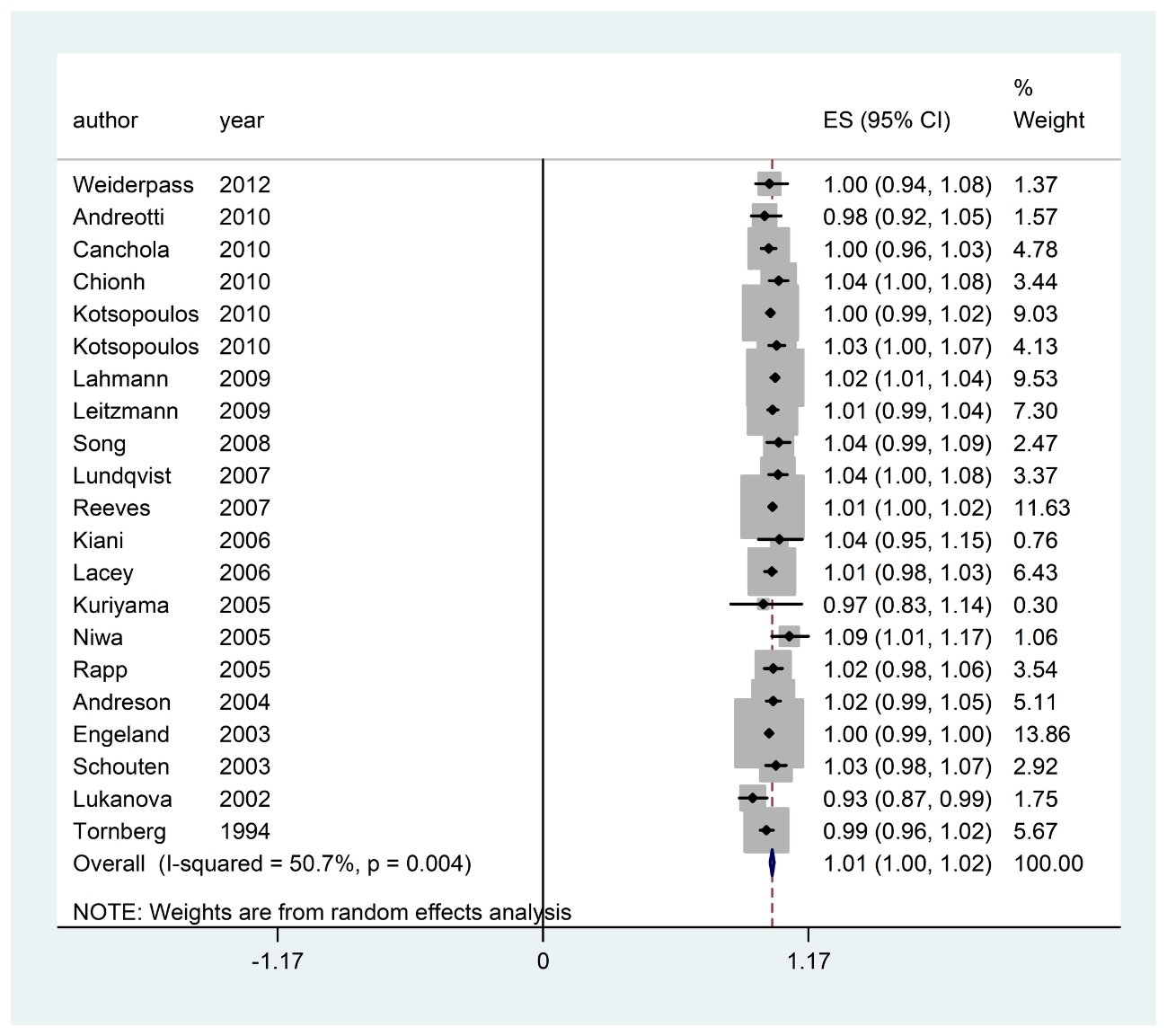


Abbreviations: ES; estimates (Relative Risk); CI, confidence interval.

Heterogeneity calculated by formula: Q = SIGMA_i{(1/variance_i)*(effect_i - effect_pooled)^2} where variance_i = ((upper limit - lower limit)/(2*z))^2

Heterogeneity chi-squared = 40.54 (d.f. = 20) p = 0.004

I-squared (variation in ES attributable to heterogeneity) = 50.7%

Estimate of between-study variance Tau-squared = 0.0001

Test of ES=0: z= 228.91 p = 0.000

**Figure 2**. Forest plot of the incidence studies of prostate cancer.


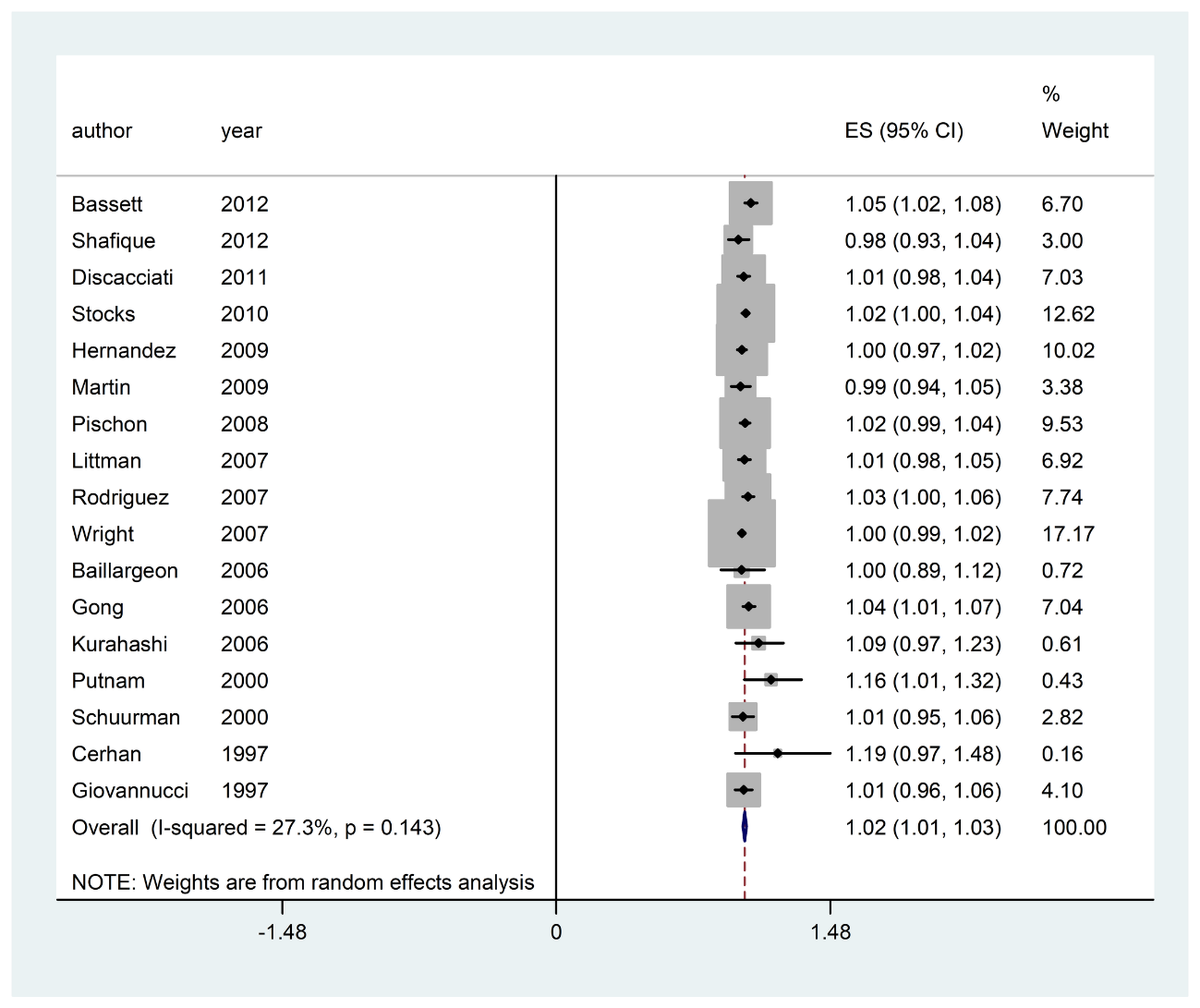


Abbreviations: ES; estimates (Relative Risk); CI, confidence interval.

Heterogeneity calculated by formula: Q = SIGMA_i{(1/variance_i)*(effect_i - effect_pooled)^2} where variance_i = ((upper limit - lower limit)/(2*z))^2

Heterogeneity chi-squared = 22.02 (d.f. = 16) p = 0.143

I-squared (variation in ES attributable to heterogeneity) = 27.3%

Estimate of between-study variance Tau-squared = 0.0001

Test of ES=0: z= 195.33 p = 0.000
